# Magnesium Sulfate in Neonatal Hypoxic–Ischemic Encephalopathy: Bridging the Gap Between Molecular Neuroprotection and Clinical Outcomes

**DOI:** 10.3390/cimb48080844

**Published:** 2026-08-20

**Authors:** Maria Ester Canepa, Federico Prefumo, Pasquale Striano, Andrea Calandrino, Luca Antonio Ramenghi

**Affiliations:** 1 Department of Neuroscience, Ophthalmology, Genetics and Mother-Child Health, University of Genoa, 16148 Genoa, Italylucaramenghi@gaslini.org (L.A.R.); 2 Obstetrics and Gynecology Unit, IRCCS Giannina Gaslini, 16147 Genoa, Italy; federicoprefumo@gaslini.org; 3 Paediatric Neurology and Muscular Disease Unit, IRCCS Giannina Gaslini, 16147 Genoa, Italy; 4 Neonatal Intensive Care Unit, IRCCS Giannina Gaslini, 16147 Genoa, Italy

**Keywords:** Hypoxic–ischemic encephalopathy, magnesium sulfate, neuroprotection, excitotoxicity, NMDA receptor, neuroinflammation, therapeutic hypothermia, translational medicine

## Abstract

Neonatal hypoxic–ischemic encephalopathy (HIE) remains one of the leading causes of neonatal mortality and long-term neurodevelopmental disability despite therapeutic hypothermia, which provides only partial neuroprotection. Among adjunctive therapies, magnesium sulfate (MgSO_4_) has emerged as one of the most biologically plausible neuroprotective agents because of its ability to modulate glutamate-mediated excitotoxicity, intracellular calcium influx, oxidative stress, neuroinflammation, and apoptotic pathways. Nevertheless, encouraging molecular and preclinical findings have not translated into consistent clinical benefit. This narrative review critically examines the translational gap between the molecular mechanisms of magnesium sulfate and its clinical performance in neonatal HIE. Evidence from experimental models, clinical studies and recent meta-analyses was integrated to identify the biological and methodological factors potentially responsible for this discrepancy. We discuss the evolving pathophysiology of HIE across the primary, latent, secondary and tertiary phases of brain injury and analyze how the timing of intervention, lesion heterogeneity and inadequate biological stratification may influence therapeutic responsiveness. Current clinical research has largely evaluated broad neurological outcomes, particularly cerebral palsy, despite the heterogeneous neuropathological substrates underlying neonatal brain injury. We argue that this strategy may dilute genuine treatment effects by grouping together distinct lesion phenotypes with different biological mechanisms. Accordingly, we propose the “Neuroprotection per Effective Therapy (NET)” framework, a conceptual translational model integrating molecular targets, experimental evidence, MRI-defined lesion phenotypes, methodological quality, and advanced statistical approaches to improve patient stratification and outcome selection. Rather than questioning the biological efficacy of magnesium sulfate itself, this review suggests that future progress will depend on aligning molecular mechanisms with clinically meaningful phenotypes. Precision-based translational strategies may ultimately allow magnesium sulfate and other neuroprotective therapies to better reveal their therapeutic effects in biologically appropriate patient subgroups.

## 1. Introduction

### 1.1. Neonatal HIE as an Unmet Clinical Challenge

Neonatal hypoxic–ischemic encephalopathy (HIE) is currently a major global challenge. The Global Burden of Disease study highlighted that in 2021 it was among the ten neurological conditions contributing to the highest age-standardized disability-adjusted life years (DALYs) [1]. Furthermore, its diagnosis could be difficult in the absence of sentinel events and radiological signs (MRI) [2,3,4]. Besides various attempts to explore the distinctive and predictive HIE pathophysiology (placental markers, changes in bilirubin, continuous glucose monitoring, protein S100, and urine markers of abnormal MRI features) [5,6,7,8,9,10], neuroprotective drugs are increasingly being investigated to reduce and prevent brain injury incidence in relation to mortality and negative neurodevelopmental outcomes [11,12,13,14].

### 1.2. Limitations of Therapeutic Hypothermia

Therapeutic hypothermia remains the standard of care. Researchers focused mainly on how to extend the benefits to neonatal populations and neuroprotective effects [15]. However, it does not necessarily protect neonates from brain injury after HIE, as described by Ghi et al. [16] despite the promising results that emerged in the TOBY trial [17,18]. The optimal timing of MRI for long-term prognostic purposes remains unclear, as reviewed by Flyger et al. [19]. Moreover, hypothermia could influence interpretation of post-therapeutic signs in MRI. For instance, Zhuang et al. [20] included routine treatment of severe neonatal HIE as a limitation of their radiomic study aiming to detect venous signature of this pathology because of hypothermia-related changes in images and metabolites.

### 1.3. Why Magnesium Sulfate?

Over the past three decades, magnesium sulfate has progressively emerged as one of the most biologically plausible neuroprotective agents. However, biological plausibility alone may be insufficient to guarantee clinical efficacy. Since HIE in term infants mirrors a progressive cascade of excito-oxidative events unfolding in the brain after an asphyxial insult, Johnston et al. recommended in 2011 to identify additional medications adjuvant to hypothermia [21]. Among neuroprotective drugs, magnesium sulfate (MgSO_4_) is considered a promising neuroprotective strategy [22], especially before maternal labor [23]. Chemically, magnesium is an ionized mineral essential to hundreds of enzymatic processes, including hormone receptor binding, energy metabolism, muscle contractility, as well as neuronal and neurotransmitter function [24], while sulfate is a crucial anion implicated in modulating exogens, polysaccharide chains of proteoglycans, cholesterol and its derivatives, and tyrosine residues of a myriad of proteins [25]. Preclinical in vivo studies in neonatal rodent models have consistently demonstrated that magnesium sulfate attenuates excitotoxic and inflammatory pathways after hypoxic–ischemic injury, thereby providing the biological rationale for its subsequent clinical evaluation [26]. However, decades of research have shown that preclinical studies should be more rigorous [27]. Experimental evidence accumulated progressively during the late 1980s [28], and throughout the 1990s, establishing magnesium sulfate as one of the earliest candidate neuroprotective agents for neonatal brain injury, despite some scientific accidents described by the group of Levene et al. [29] in which neonates received 150 mg of MgSO_4_, which was inadvertently administered as a 250 mg bolus when diluting it in water. Importantly, the publication by Levene et al. laid the foundation of the international network for neuroprotective evaluation after severe birth asphyxia [30] in 1999. Maternal antenatal administration of magnesium sulfate prior to preterm delivery (before 32 or 34 weeks) demonstrated a reduction in cerebral palsy (CP) incidence (a motor condition) in trials and meta-analyses [31,32,33,34]. Notwithstanding these encouraging results [35,36], current data are suffering from selective reporting bias and pathophysiology misalignments: magnesium sulfate administration is not sufficient to provide complete neuroprotective care according to survey studies [37], and its effect on the reduction in intraventricular hemorrhage (IVH) is uncertain [38]. Furthermore, pathophysiological evidence demonstrates that CP could result from diverse brain injury per phenotype and epidemiological time-dependent incidence (hemorrhagic lesions like intraventricular and cerebellar hemorrhage; ischemic lesions like periventricular leukomalacia and white matter injury) [39,40,41,42,43,44]. For instance, Calandrino et al. [42] reported cerebellar disruptions in preterm neonates with germinal matrix and intraventricular hemorrhages. Conversely, antenatal magnesium sulfate does not accelerate white matter maturation in preterm neonates at term-equivalent age (TEA) [45], and in a further Canadian study, functional connectivity is enhanced at TEA [46]. Hence, these groups of lesions lead to white matter necrosis [47,48]. The inconsistencies in antenatal magnesium sulfate effects may reflect inadequate biological stratification [49]. Accordingly, a target-unified magnesium therapy could not be appropriate without considering molecular pathways and pathophysiological patterns. Hence, the neuroprotective effect of antenatal magnesium sulfate in early preterm newborns cannot automatically be extrapolated to term newborns with HIE. Furthermore, the combination of multiple drugs (erythropoietin, magnesium sulfate, melatonin, topiramate, xenon, and darbepoetin alfa) cannot reduce mortality with low-quality evidence [50]. In the recent literature, AI/ML and bio-orthogonal chemistry for engineering smart soft polymeric nanocarriers highlight opportunities in closed-loop discovery and personalized nanomedicine [51]. To the best of our knowledge, no review has comprehensively explored the gap between the molecular biology of magnesium sulfate and HIE pathophysiology.

### 1.4. Aim of the Review

A narrative review was considered the most appropriate approach for critically examining why strong molecular neuroprotective effects have not translated into consistent clinical benefit, as this question requires the integration of molecular, preclinical, and clinical evidence rather than quantitative synthesis alone [52] considering both preterm and term neonates as well as antenatal administration of magnesium sulfate. For this purpose, a literature search was performed in PubMed/MEDLINE, Cochrane, EMBASE, and Scopus using combinations of the terms “magnesium sulfate”, “magnesium sulphate”, “hypoxic-ischaemic encephalopathy”, “neonatal encephalopathy”, “perinatal asphyxia”, “neuroprotection”, and “therapeutic hypothermia”. Priority was given to experimental studies, randomized clinical trials, systematic reviews, meta-analyses, and recent translational literature relevant to neonatal HIE. Reference lists of key articles and recent systematic reviews were also screened to identify additional relevant studies.

## 2. Pathophysiology of Hypoxic–Ischemic Brain Injury

### 2.1. Primary Energy Failure

**Figure 1:**
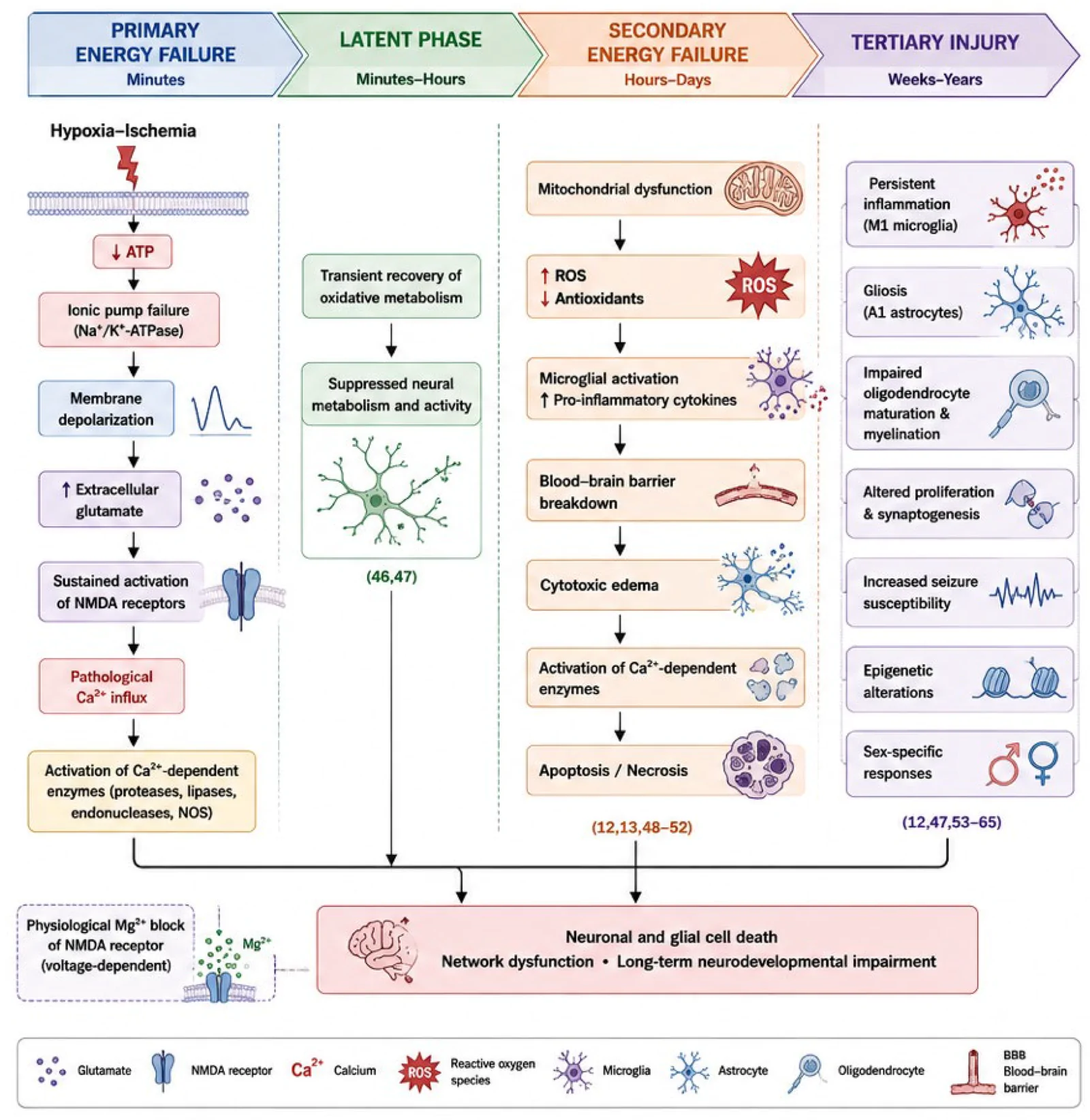
Molecular cascade of neonatal hypoxic–ischemic brain injury [12,13,46,47,48,49,50,51,52,53,54,55,56,57,58,59,60,61,62,63,64,65].

The pathogenesis of hypoxic–ischemic injury is complex and multifactorial, focusing primarily on cellular and molecular mechanisms [53]. To better understand the neuroprotective role of magnesium sulfate, it is important to describe the molecular pathophysiology of HIE and its links with MgSO_4_ pharmacodynamics [3,13,54,55]. Hypoxic–ischemic injury is characterized by three stages: latent, secondary, and tertiary [56] as illustrated in Figure 1. Specifically, during the primary phase of hypoxic–ischemic injury, depletion of intracellular ATP disrupts ionic homeostasis, causing membrane depolarization and excessive extracellular accumulation of glutamate. Sustained activation of NMDA receptors results in pathological calcium influx, which initiates multiple downstream cascades including mitochondrial dysfunction, oxidative stress, activation of calcium-dependent enzymes, and programmed cell death. These processes constitute the core of glutamate-mediated excitotoxicity and represent one of the earliest therapeutic targets in neonatal HIE. Since Mg^2+^ physiologically blocks the NMDA receptor channel in a voltage-dependent manner, this pathway represents one of the earliest and most biologically plausible therapeutic targets in neonatal HIE. If excitotoxicity predominates, NMDA blockade may theoretically exert greater benefit than in infants whose injury is primarily driven by inflammation or delayed repair failure.

### 2.2. Latent Phase

During profound hypoxia-ischemia, brain cells may die, although some could recover oxidative metabolism. This period is defined as “latent,” in which neural metabolism and activity are suppressed [57]. Thereafter, after moderate to severe hypoxia-ischemia, this transient recovery is followed by a 6 h phase of secondary deterioration, with delayed seizures, failure of mitochondrial function, cytotoxic edema, and cell death over 72 h.

### 2.3. Secondary Energy Failure

Progression of acute cerebral hypoxia-ischemia to an inflammatory response is a major contributor to the pathophysiology of neonatal brain injury [58]. As portrayed by Li et al. [58], hypoxia-ischemia elicits an intravascular inflammatory cascade. Subsequently, the activation of resident immune cells and the cerebral infiltration of peripheral immune cells in response to cellular damage in the brain parenchyma amplify the effects of the ongoing inflammation. Thus, this protracted neuroinflammation brings about a secondary brain tissue injury. Indeed, the secondary energy failure could be generated by multiple neurotoxic phenomena like oxidative stress, mitochondrial dysfunction, neuroinflammation, and apoptosis [12,13,59]. Specifically, oxidative stress is a crucial dimension for detecting potential brain susceptibility to neural damage [60,61]. To demonstrate how it is important to consider a multisystem approach in neuroprotective strategies—including MgSO_4_ administration—the crosstalk between neonatal lung, heart, and brain is linked to apoptosis, glutamate signaling, oxidative stress, breakdown of the blood–brain barrier, and pro-inflammatory cytokines, which could be interconnected with an inflammatory pulmonary state and low cardiac output [62].

### 2.4. Tertiary Injury

Tertiary injuries could occur in the presence of persistent inflammation and impaired repair mechanisms, leading to neonatal seizures [3,12,57]. Some outcomes include sensitization to inflammation or injury, increased seizure susceptibility, persistent inflammation and gliosis, impaired oligodendrocyte maturation and myelination, altered proliferation and synaptogenesis, and epigenetic alteration [63]. This phase could last between weeks and years after perinatal injury [57]. Although rehabilitation is one step of the main therapeutic path fostering neuroplasticity [64], current evidence is still limited because of small sample sizes in the majority of studies. In this sense, intervention timing during tertiary injury is crucial for preventing further lesions. Davidson et al. [57] described the underlying cellular phenomena in detail. Largely thanks to neurodegeneration models, endogenous brain plasticity could be impaired by protracted pro-inflammatory activation (M1) of microglia [65,66], and sustained activation of astrocytes (A1), causing gliosis. These phenomena are opposed to neuroprotective and boost repair activation (M2 and A2, respectively for microglia and astrocytes) [67,68]. Beyond the increasing diffusion of pro-inflammatory factors damaging neighboring cells and endogenous plasticity mechanisms, M1 microglia cannot adequately perform physiological neurodevelopmental functions (i.e., synaptic pruning and control of synaptic functioning) [69]. Notably, some drugs may enhance M2 over M1 activation, like pioglitazone, metformin, and resveratrol [70,71,72,73]. Interestingly, evidence is increasingly developing on the conditioning power of inflammation on the integrated stress response in male fetal mice. There is emerging evidence that inflammation can affect the integrated stress response only in male fetal mice. Deterring this response improved neurobehavioral outcomes in male but not female offspring [74]. Therefore, possible sex-specific effects might modulate the inflammation process in the tertiary phase. A further study in *Nature* [75] highlighted that pregnant mice that had been colonized with mouse commensal segmented filamentous bacteria or human commensal bacteria that induce intestinal TH17 cells were more likely to produce offspring with Maternal Immune Activation (MIA)-associated abnormalities. Indeed, small intestine dendritic cells from pregnant, but not from non-pregnant, females secrete IL-1β, IL-23 and IL-6 and stimulate T cells to produce IL-17a upon exposure to MIA. Overall, these findings suggest that gut commensal bacteria with a propensity to induce TH17 cells may increase the risk of neurodevelopmental disorders in the offspring of pregnant mothers undergoing immune activation owing to infections or autoinflammatory syndromes. MIA could partially explain why the signaling channels could be impaired after exposure to inflammation or pathogens during gestation [76]. As reviewed in 2026 by She et al. [77], inflammation and oxidative stress induced by activated microglia can directly damage dopaminergic neurons, inhibiting dopamine synthesis, reuptake, and receptor activity. Enhanced microglial phagocytosis inhibits dopamine axon extension. These long-lasting effects of microglial perturbations may be driven by early life stress-induced epigenetic reprogramming of microglia. Indirectly, early life stress may influence microglial function through various pathways, such as astrocytic activation, the hypothalamic–pituitary–adrenal axis, the gut–brain axis, and maternal immune signaling. Additionally, McLeod et al. [78] reported that while MgSO_4_-treated male human preterm neonates have poor cognitive outcomes compared with untreated male neonates, female neonates have significantly improved cognitive outcomes compared with untreated female neonates after antenatal magnesium sulfate administration. These findings support the hypothesis that sex may represent an important biological modifier of neuroprotective responses and underline the need for adequately powered clinical studies specifically designed to detect sex-specific treatment effects. Accordingly, drug neuroprotection might be tailored according to the timing and phase of hypoxic–ischemic injury with the available and evidence-based therapies [56].

## 3. Molecular Basis of Magnesium Sulfate Neuroprotection

Analogously to adult cohorts, N-methyl-D-aspartate (NMDA) receptors are involved in the excitotoxicity induced by hypoxia-ischemia in HIE. GluN2A- and GluN2B-containing NMDA receptor subtypes have distinct roles in HIE pathophysiology [54]. MgSO_4_ modulates immunological activity [79]. Indeed, it inhibits cellular calcium influx and excitatory amino acid release in neurons via blockade of the NMDA-receptor channel, which is provided by a magnesium-dependent calcium gate [80,81]. As described by Nowak et al. [81], the properties of Mg^2^^+^-free solutions, L-glutamate, L-aspartate, and NMDA open cation channels are voltage independent. When Mg^2+^ is present, the probability of channel opening decreases as the single-channel currents at resting potential become fragmented. Hyperpolarization increases both effects, sloping the I-V relationship of the glutamate response negatively. The voltage dependence of the NMDA receptor-linked conductance may be derived from the voltage dependence of the Mg^2+^ block. Accordingly, its interpretation does not necessarily imply an intramembrane voltage-dependent gate.

Reviewing the most recent literature, Okazaki et al. concluded that combining hypothermia and further medications such as erythropoietin and melatonin is becoming an increasingly popular approach for neonates with perinatal asphyxia [82]. However, magnesium sulfate in combination with hypothermia did not reduce mortality in a randomized controlled trial in India [83].

## 4. Clinical Evidence in Neonatal HIE

### 4.1. Early Pilot Studies

Neuroprotective drugs play a pivotal role in neonatal care to prevent brain injuries both in term and in preterm neonates [84,85]. Caffeine and magnesium sulfate are considered fundamental in neuroprotective pharmacology, respectively, for myometrial contractility and apnea of prematurity [86]. Despite their potential clinical benefits, NICU-to-NICU administration varies greatly, and their efficacy could be limited [86]. Pilot studies aimed to assess the safety and feasibility of administering neuroprotective drugs in HIE neonates in multiple combinations (erythropoietin, magnesium sulfate, and hypothermia) [87]. Regarding magnesium sulphate, a prospective, longitudinal, placebo-controlled trial conducted in India found that postnatal magnesium sulphate treatment improved neurological outcomes at discharge in term neonates with severe perinatal asphyxia [88]. Although no adverse effects are associated with magnesium sulfate according to a systematic review of randomized trials set in low- and middle-income countries [89], few studies reported an increase in neonatal apnea requiring ventilation, such as in the trial by Bhat et al. [88], who identified two infants with this condition. Nonetheless, more robust, multicentric randomized controlled trials are needed to confirm the potential of the broad spectrum of neuroprotective drugs, including magnesium sulfate. Indeed, multicenter randomized, double-blind, controlled trials such as the PENUTS study [90] in extremely preterm neonates showed no significant differences between erythropoietin and placebo in the rates of retinopathy of prematurity, intracranial hemorrhage, sepsis, necrotizing enterocolitis, bronchopulmonary dysplasia, and death or in the frequency of serious adverse events; moreover, high-dose erythropoietin treatment administered to extremely preterm infants from 24 h after birth through 32 weeks of postmenstrual age did not result in a lower risk of severe neurodevelopmental impairment or death at 2 years of age.

### 4.2. Recent Meta-Analyses and Systematic Reviews

Meta-analysis represents one of the highest tiers of the evidence pyramid [91] together with randomized trials and systematic reviews. In this review, the evidence on magnesium sulfate is fragmented into multiple categories of methodological strength connected with statistical significance. Analyzing 20 randomized trials (total sample size: 1485), Gowda et al. [92] showed that magnesium sulfate could improve neurological outcomes in settings where therapeutic hypothermia is not available, but it does not reduce mortality. By contrast, the meta-analysis by Crowther et al. on five trials (total sample size: 5493 women at risk of preterm birth and 6131 babies), Ref. [34], shows that antenatal magnesium sulfate given prior to preterm birth for fetal neuroprotection prevents CP and reduces the combined risk of fetal/infant death or CP, regardless of the reason for preterm birth, with similar effects across a range of preterm gestational ages and different treatment regimens. Such comparisons may overlook important biological determinants, including lesion nature (i.e., severity grade of germinal matrix-intraventricular hemorrhage, significant cerebellar hemorrhage [43], and periventricular leukomalacia) and timing [93,94], developmental stage, concomitant neuroprotective therapies, and coexisting neonatal morbidities. Taken together, these findings suggest that differences in patient population, developmental stage, timing of magnesium sulfate administration, and outcome selection may explain much of the apparent inconsistency across the literature. Accordingly, these meta-analyses are an illustrative example of what we define as the “Ping-Pong effect”, i.e., the recurrent shift in statistical significance from one clinical endpoint to another across successive randomized trials and meta-analyses, resulting in inconsistent perceptions of therapeutic efficacy despite a stable biological rationale. Indeed, network meta-analyses underscored that no combination therapy can reduce mortality, seizures, or abnormal brain imaging findings in neonatal hypoxic–ischemic encephalopathy. Still, hypothermia combined with melatonin may reduce adverse neurodevelopmental outcomes in neonates with HIE, although the overall quality of evidence remains low [50].

## 5. The Translational Paradox

### 5.1. Why Does Molecular Efficacy Not Translate into Clinical Benefit?

Magnesium sulfate literature is a textbook example of disappointing efficacy in clinical research despite preclinical evidence. Regarding term neonates with hypoxic lesions, Gressens et al. [95] argued a misalignment between in vitro models and in vivo phenotype. In international research on HIE, mouse studies underscored the evidence of molecular and excito-oxidic pathways, but they failed in clinical trials on human neonates [96] despite the neonatal brain being susceptible to excitotoxicity because of its immaturity [23]. In addition, neonatologists do not know when a hypoxic–ischemic event could occur in human neonates rather than in mice [97]. Recent observational studies have progressively incorporated biologically grounded hypotheses and clinically defined high-risk populations, partially addressing the translational gap. However, patient stratification still relies predominantly on clinical characteristics rather than molecular endotypes or biomarker-guided precision approaches [98].

### 5.2. The Neuroprotection per Effective Therapy (NET) Framework: A Conceptual Proposal for Translational Neonatal Neuroprotection

**Figure 2:**
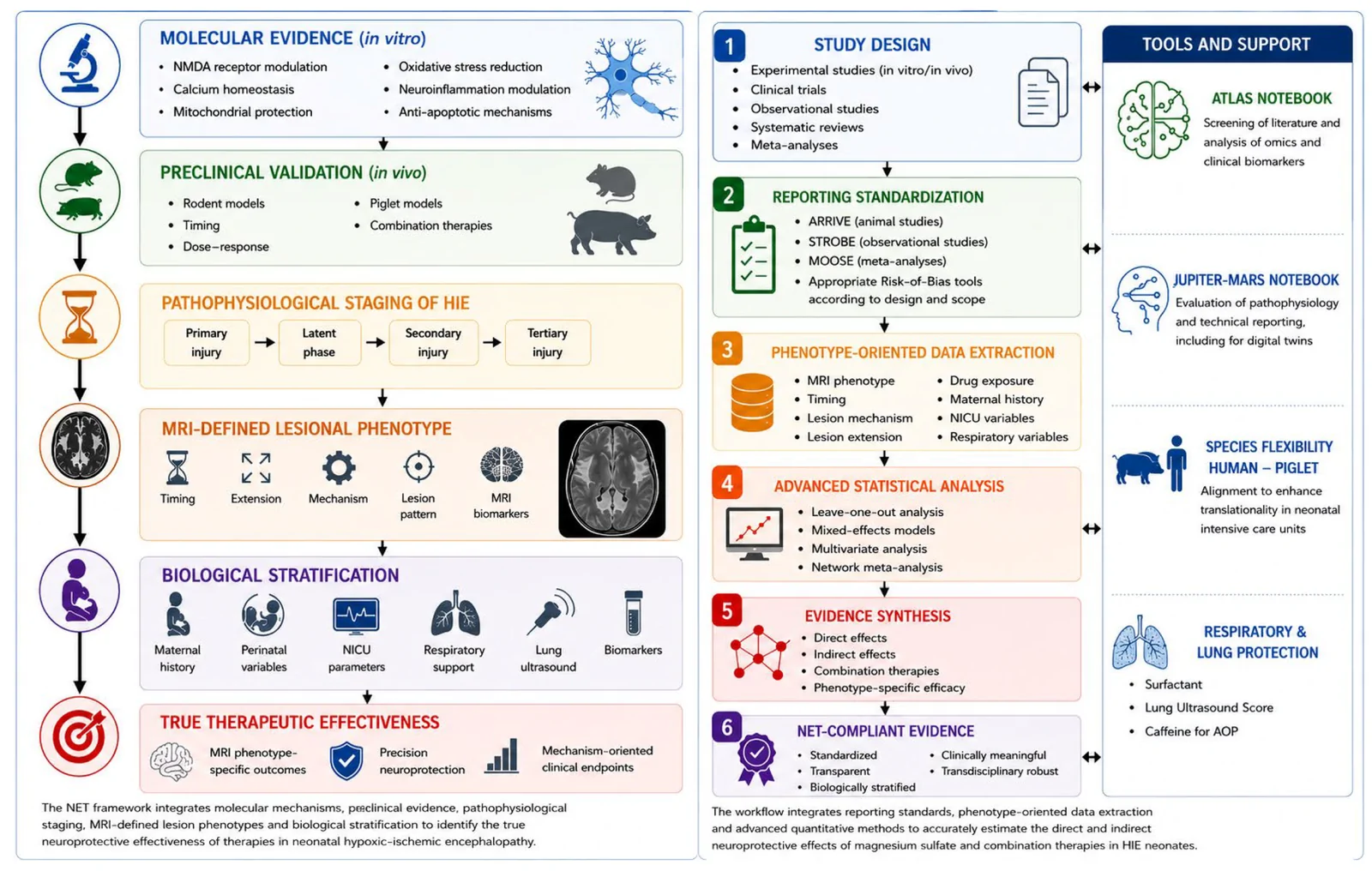
NET framework from molecular mechanisms to clinically meaningful neuroprotection (**left**); NET-compliant methodology for translational neonatal neuroprotection research (**right**).

Considering the neuroprotective drug pitfalls underscored by Gressens and Galinsky [49,95], we propose the NET framework (Neuroprotection per Effective Therapy) in Figure 2, consisting of a vectorial structure from in vitro evidence to in vivo MRI phenotype as clinical endpoints, based on MRI-defined lesion phenotypes per timing, extension, and mechanism using appropriate statistical methods to quantify authentic drug effectiveness. If feasible, omics analyses could be included as molecular biomarkers, as we will be describing in Section 7. NET is a hypothesis-generating proposal for enhancing precision neuroprotection, integrating imaging, clinical biomarkers, and biological phenotype, aimed at helping neonatologists and healthcare providers to guide treatment decisions. Future evidence synthesis should incorporate biologically informed lesion phenotypes and sensitivity analyses (e.g., leave-one-out procedures) to better identify studies contributing disproportionately to heterogeneity (effect size and quantitative evidence synthesis). Further studies should integrate clinical, imaging, and biological variables within multivariable analytical frameworks [99]. These approaches may clarify whether magnesium sulfate exerts differential effects according to lesion phenotype, treatment timing, respiratory status, and concomitant neuroprotective interventions. Network meta-analysis may further help compare combination strategies involving hypothermia and adjunctive therapies [50,100,101,102]. In this way, researchers could also conduct magnesium sulfate studies in alignment with cross-species translation between human and animal models, thereby shaping the translationality of neonatological research [103]. Studies implementing the NET framework should adhere to internationally accepted reporting standards according to study design (e.g., STROBE, MOOSE and ARRIVE) [104,105,106], ensuring methodological transparency and reproducibility. The clinical dimensions (Risk of Bias and reporting assessment) should consider the appropriate checklists according to the design and scope. In such a manner, methodology and reporting may become more standardized and transparent to the benefit of pharmacological research on MgSO_4_, overcoming the limitation that some neural anti-inflammatory therapy studies did not clearly state whether they controlled for a number of potential biases [107]. Technological instrumentation should be tailored to scientific feasibility, economic sustainability, and the research question. Basak et al. [51] reviewed Artificial Intelligence/Machine Learning pipelines and bio-orthogonal chemistry for engineering smart soft polymeric nanocarriers as opportunities in closed-loop discovery and personalized nanomedicine. However, translational barriers emerged regarding datasets, interpretability, reproducibility, manufacturability, and regulation. This group suggested providing standardized and shareable datasets, automation-compatible modular chemistry, interpretable predictive models with uncertainty reporting, and translational criteria that include manufacturability, stability, and regulatory feasibility. In pediatrics, open datasets are challenging due to data protection law and under-representation of children and neonates in international imaging datasets, radiomics studies, and Machine Learning/deep learning pipelines [108,109,110].

### 5.3. Failure of “One Drug-One Mechanism” Approaches

HIE is a multifactorial disease as much as magnesium sulfate is a multitarget drug. We propose that the magnesium sulfate effect is diluted into a major outcome-container named “Cerebral Palsy” (CP), leading to a more prominent “pathology of evidence” contaminating both primary study results and systematic reviews. By “pathology of evidence”, we refer to the systematic distortion of the scientific literature caused by data fishing, p-hacking, cherry-picking, publication bias, enrolment bias, and selective outcome reporting, ultimately generating an apparently contradictory body of evidence that may distort conclusions regarding therapeutic efficacy in clinical trials and meta-analyses [111,112,113,114,115,116,117,118] that ultimately drive conclusions about any intervention efficacy [119] in trials and meta-analyses. Therefore, quantifying and mitigating cherry-picking represents an important step towards improving the reliability of neuroscientific research [120]. With NET, we suggest considering the lesional pattern according to onset timing and imaging signs. Analogously, Brenner et al. analyzed the trial CRASH-3 on the neuroprotective effect of tranexamic acid (TXA) in adult intensive care patients with head traumas. Crucially, TXA effects were diluted into a further outcome-container, that is, all-cause mortality reduction. Similarly to our approach, Brenner et al. underscored how effective neuroprotection was dependent on TXA administration timing, considering the underlying mechanism of hemorrhage.

### 5.4. Tools for Applying NET Principles

To perform a preliminary screening of literature in Artificial Intelligence (AI) applied to imaging, radiomics, and clinical biomarkers (if necessary), we have developed the ATLAS Notebook. Having been benchmarked on multiple papers on adult oncology, pediatric oncology, and neonatology, it extracts directly from Portable Document Format files relevant keywords associated with standard checklists. Additionally, to evaluate pathophysiology reporting even for digital twins (virtual replicas of organs, patients, and biological systems to inform clinical decisions) [121], the JUPITER-MARS Notebook [122] could assist healthcare researchers to evaluate both pathophysiology and technical reporting analogously to the ATLAS Notebook. We acknowledge the exploratory nature of these tools, necessitating further prospective validation, beyond human audit of their outputs.

## 6. Towards MRI Biomarkers of Neuroprotective Drug Response

This review has depicted how molecular pathways are helpful for understanding HIE pathophysiology and magnesium sulfate effects. However, as previously remarked, the current contribution is not sufficient to provide a comprehensive knowledge of its neuroprotective role. Furthermore, omics and molecular biology labs are not available in every hospital. Therefore, a more feasible approach is needed to balance current evidence and medical research instruments. For instance, imaging could be a potential biomarker source rather than a monitoring tool alone. Radiomics is one potential strategy to study pathology and pathophysiology of HIE [20,123,124,125]. Additionally, ComBat-based harmonisation of multi-site MRI data [126,127] represents a further strategy to both overcome scanner bias across research centers in MRI diffusion tensor imaging (DTI) and enlarge neonatal cohorts to analyze neuroprotective drug effects [128]; this strategy is being employed in the ACUMEN study [129] on melatonin administration for moderate and severe HIE neonates treated with hypothermia.

## 7. Towards Molecular Biomarkers of Neuroprotective Response: The Omics Contribution

As the radiomics approach could be more generalizable when combined with a molecular analysis (genomics, etc.), as indicated by the specific Radiomic Quality Score item [130], molecular biomarkers should not simply predict prognosis, but identify biological endotypes potentially responsive to specific neuroprotective interventions integrated with the preexisting radiological and clinical features [131] as previously seen in neonatal mice [132].

Whilst proteomics, lipidomics, transcriptomics, and epigenomics are promising and less validated for clinical use, metabolomics remains the most mature approach despite reduced sample size, standardization, and external validation per study [133]. Based on the previous research [134,135], these studies identified alterations consistent with mitochondrial energetic deficit, oxidative stress, amino acid metabolism, purines, acylcarnitines, and lipids. Some models combined blood cord or integrated with plasma and urine metabolites, mass spectrometry, and MRI and other clinical parameters to improve HIE or further brain injury stratification on MRI, as shown by O’Boyle et al. and Locci et al., respectively [136,137]. Particularly, Locci et al. revealed lactate, succinate, hypoxanthine, amino acids, and acylcarnitines as biomarkers; nonetheless, this methodology is not replicable across centers. In order to capture the metabolomic signature varying per the HIE-induced brain injury severity [138], underlying neurological processes that may not necessarily be reflected on MRI [139], and the dynamic nature of the metabolomic signature of being modified across the first two hours of life by treatment and disease [135], one replicable, feasible, and non-invasive analysis is on the urine samples. Studies by Solevåg et al. on oxidative stress and Pineiro et al. concerning HIE metabolome underscored, respectively, that this approach could be a serial monitoring strategy before and after hypothermia treatment without serial blood samples [140,141]. As such, lipidomics could be a promising integrative framework with metabolomics. Indeed, Nixon et al. [142] identified lipidomic signatures on dried blood spots in hypothermia-treated HIE neonates, as they may provide biomarkers related to membrane and myelination damage.

## 8. Conclusions

Magnesium sulfate remains one of the most biologically plausible neuroprotective agents in neonatal HIE. However, the discrepancy between molecular efficacy and clinical outcomes highlights the complexity of translational neuroprotection and underscores the need for precision-based therapeutic strategies.

## Data Availability

No new data were created or analyzed in this study. Data sharing is not applicable to this article.
